# Clinical and demographic characteristics of children with familial mediterranean fever in Central Anatolia

**DOI:** 10.1186/1546-0096-9-S1-P11

**Published:** 2011-09-14

**Authors:** Onur Akin, Erkan Demirkaya, Adem Polat, Ezgi Deniz Batu, Yusuf Tunca, Cengizhan Acikel, Harun Peru, Betul Sozeri, Ismail Dursun, Hakan Poyrazoglu, Faysal Gok, Seza Ozen

**Affiliations:** 1 Gülhane Military Medical Academy, Pediatric Nephrology & Rheumatology Unit, Ankara, Turkey; 2 Hacettepe University Medical School, Pediatric Nephrology & Rheumatology Unit, Ankara, Turkey; 3 Gulhane Military Medical Faculty, Department of Medical Genetic, Ankara, Turkey; 4 Gulhane Military Medical Faculty, Department of Public Health, Division of Epidemiology, Ankara, Turkey; 5 Selcuklu University, Paediatric Nephrology & Rheumatology Unit, Konya, Turkey; 6 Ege University Medical School, Pediatric Nephrology & Rheumatology Unit, İzmir, Turkey; 7 Kayseri Education and Research Hospital, Kayseri, Turkey; 8 Erciyes University Medical School, Pediatric Nephrology & Rheumatology Unit, Kayseri, Turkey

## Objective

This study investigates the clinical and demographic characteristics of Turkish familial Mediterranean fever (FMF) patients and relationship between these characteristics and genotype.

## Methods

115 girls and 143 boys were included in this study. The clinical data of patients were collected from six centers. Genetic analysis was performed using polymerase chain reaction and restriction endonuclease digestion methods to detect the presence of eight FMF gene mutations.

## Results

The mean age of disease onset was 5.1 ± 3.6 years and the mean age at diagnosis was 7.3 ±3.1 years. The mean number of attacks per year was 7.7 ± 7.8, the mean duration of attacks 1.7 ± 1.6 days. The most common clinical manifestations were fever (91.6%) and abdominal pain (90.3%). The other manifestations were arthralgia (44.1%), arthritis (18.6%), headache (17.3%), myalgia (15.6%), vomiting (11%), chest pain (6.8%), splenomegaly (5.5%), diarrhea (5.5%) and erisipelas like eritema (3.4%). Amyloidosis (1.8%) and proteinuria (1.8%) were also determined. Most of patients had compound heterozygote genotype (n=86, 28.6%) and the most common homozygote mutation was M694V homozygosity (n=65, %21.6). The frequency of headache in the patients with homozygote M694V mutation (n=18, %29.5) and the frequencies of arthritis in patients with homozygote E148Q (n=1, %33.3) and M694V mutations (n=16, %26.2) were significantly higher than the patients with other mutations (p<0.05, for all of them).

## Conclusion

The results of this study supported the hypothesis that mutation types seem to determine the susceptibility to some clinical features development. With the determination of new mutations, further studies are warranted to investigate relationship between genotype and phenotype in larger populations.

